# Mitigation of cascading failures in complex networks

**DOI:** 10.1038/s41598-020-72771-4

**Published:** 2020-09-30

**Authors:** Alex Smolyak, Orr Levy, Irena Vodenska, Sergey Buldyrev, Shlomo Havlin

**Affiliations:** 1 Department of Physics, Bar-Ilan University, 52900 Ramat-Gan, Israel; 2 Department of Administrative Sciences, Metropolitan College, Boston University, 1010 Commonwealth Avenue, Boston, MA 02215 USA; 3 Department of Physics, Yeshiva University, 500 West 185th Street, New York, 10033 USA

**Keywords:** Complex networks, Phase transitions and critical phenomena, Computational science

## Abstract

Cascading failures in many systems such as infrastructures or financial networks can lead to catastrophic system collapse. We develop here an intuitive, powerful and simple-to-implement approach for mitigation of cascading failures on complex networks based on local network structure. We offer an algorithm to select critical nodes, the protection of which ensures better survival of the network. We demonstrate the strength of our approach compared to various standard mitigation techniques. We show the efficacy of our method on various network structures and failure mechanisms, and finally demonstrate its merit on an example of a real network of financial holdings.

## Introduction

Since complex systems emerged as a prolific area of applied studies around the turn of the century^1–3^, network science methodologies have been successfully developed and used to better understand many domains. A recurring theme, going back almost as far as the field itself, is the propagation of information of different types over complex networks. Examples include epidemic spreading, opinion formation, and failure propagation. Biological, social, computer and other networks all exhibit spreading dynamics through different mechanisms resulting in rich behavior. In the context of computer and other physical networks, such as power grids, financial systems, social networks and communication systems, cascading failures have been extensively studied^4–10^. What makes the study of cascading failures so important is the fact that an actual catastrophe, such as infrastructure collapse, global epidemic or a financial meltdown may happen seemingly without warning, starting from a very small failure.

Determining whether the system is in a state where small, local failures could spread globally, and lead to a system-wide collapse may be impossible without precise knowledge of system variables and parameters. This precise knowledge is rarely attainable in real life dynamics. That knowledge may include, depending on the setting, failure mechanisms, the global connectivity patterns, network-wide degree distributions and more. Interdependent networks^4^ increase the complexity of the analysis by conditioning survival on dependence between different networks. As a concrete example, network science proved to be a highly appropriate approach to study financial systems after the 2008 housing bubble collapse. Decline in housing prices in the United Stated led to a global credit crunch, transmitted via complex financial instruments and tight coupling between institutions, increasing financial system vulnerability for a prolonged period of time. Soon after bursting of the real estate bubble, which brought down many financial institutions, research highlighted the importance of network relations between these institutions that led to the propagation of failure (see e.g.,^11–15^). Research conducted in complexity science analyzing contagion, centrality and impact of failure of financial institutions aims to aid financial regulation and monitoring by identifying the network aspects of financial system uncertainty^5^.

When considering network failure models, we need to take into account several important parameters. These parameters include reversibility of failure (whether or not a node recovers after failure); the way a node’s immediate neighborhood affects its own failure likelihood; and whether or not global connectivity, such as belonging to the giant component of a network plays a role, to name a few. Other properties characterize the network structure as a whole. It may be simple, weighted, bipartite, multilayer, interdependent, with various degree distributions, correlations, communities, clustering properties and more^16,17^. An exhaustive analysis of all variants is impossible, so we choose relatively simple but broadly applicable models with several variants and offer non-trivial insights on mitigating cascading failures based on local neighborhoods information.

**Figure 1:**
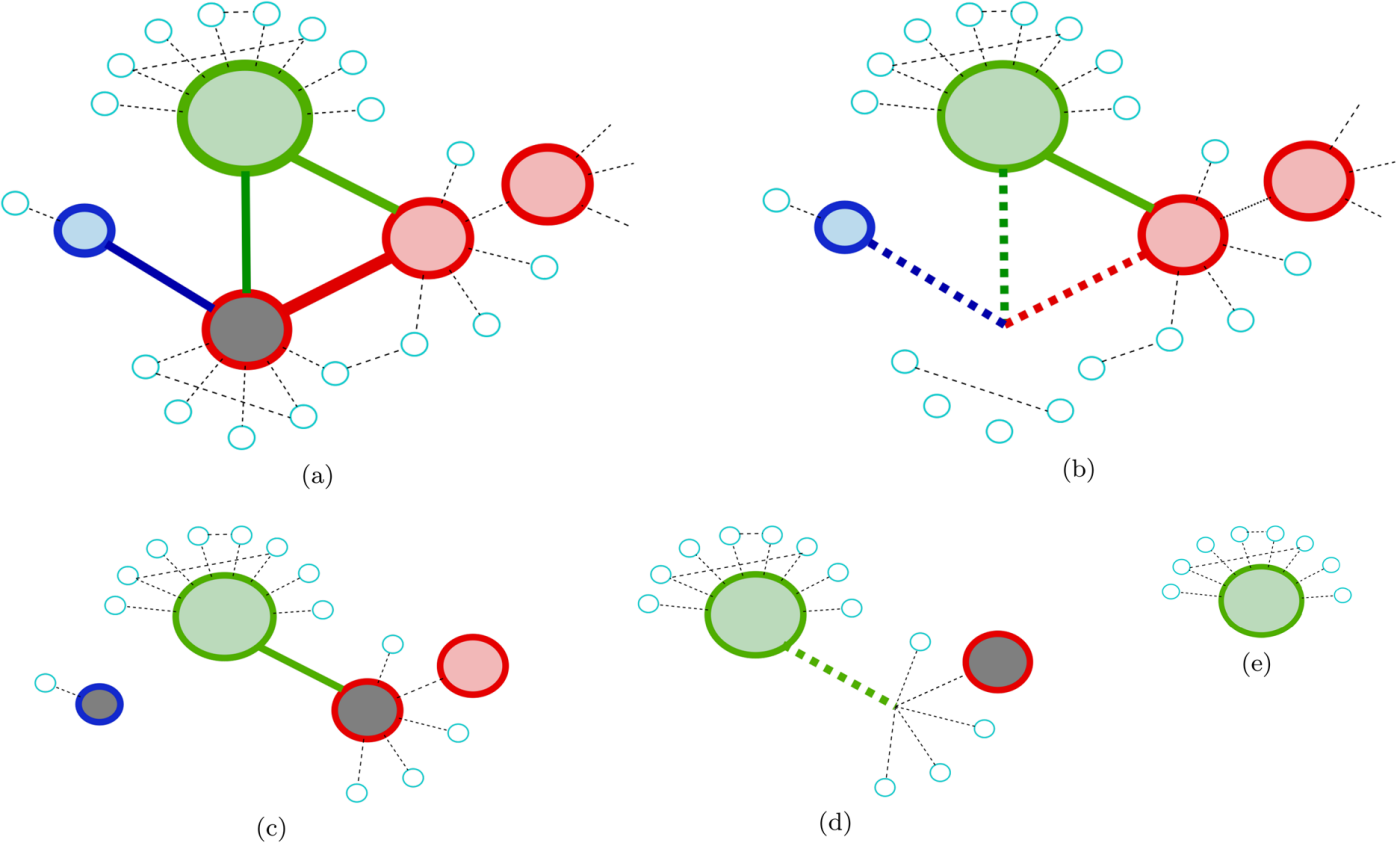
Description of the fractional cascade process, colors illustrate the immunization algorithm described in “Mitigation strategy”. (**a**) The gray node is impacted and will be removed; (**b**) when removed, all its edges disappear as well; (**c**) here the important difference between the blue (low degree), green (high degree) and red (medium degree) nodes is highlighted. The blue node has only one additional neighbor, so its failure does not exacerbate the cascade. The green node is of a high degree, so it is not affected by the single neighbor’s failure. The red nodes, however, are vulnerable with respect to a threshold failure perspective, but also have a large number of neighbors that will be affected by their failure. They are the ones enhancing the cascading process; (**d**,**e**) show the termination of the failure on the toy model example with only the high-degree node (green) surviving the process.

Cascading processes on networks may either be the goal, such as in the context of marketing and advertising, where the target may be to optimally disseminate information across networks^6–9^, or an adverse result (Fig. 1), e.g. in finance, health care or infrastructures, where cascading process of failure or disease spreading is detrimental, and should ideally be avoided, mitigated or stopped.

The literature on the subject is diverse, taking multiple different approaches^18–24^. Those range a broad-spectrum, descriptive to fine-grained calculations and algorithms. The domain of applicability of both cascades and mitigation is very broad. Protection methodologies may be specific (i.e. targeting concrete nodes or edges^18,23^) or statistical (that is, selecting a fraction of nodes conforming to some condition^20,24^), broad^19,24^ or case-specific^21,23^, probabilistic or deterministic. Some aim at identification of important nodes, others wish to maximize system survival. A complimentary approach is that of healing or recovery^25,26^.

In this paper we analyze several variants of network structures and failure methods mentioned above. We aim to propose a simple, bottom-up, easily implemented model for efficient mitigation of cascades on a given network. We show that the knowledge required for such model is limited to node’s nearest neighbors, ignoring higher-order connectivity even in a non-treelike structure (Fig. 1). The resulting mitigation strategy is globally very effective. We show that our approach is appropriate for a wide variety of network topologies and various failure mechanisms. Using financial cascading failure as an example, we aim to identify nodes, representing financial institutions, that we need to protect in order to keep the network in a connected, functional state. To accomplish this task, we inspect the behavior of simple network models having different degree distributions, bipartite networks as well as interdependent networks. Finally, we test our process and show its effectiveness on an actual bank-asset data-set, exploring failure mitigation of a real world network by applying our methodology. This is important not only as a demonstration of the model’s strength in real-life applicability, but also as an example of a simple model that can be extended to more complex settings and still perform very well.

Our approach may be applied to a broad range of systems for prevention and mitigation of cascading failures. Specifically, in relation to financial stability, it may help regulators to better protect the system and mitigate future collapse.

**Figure 2:**
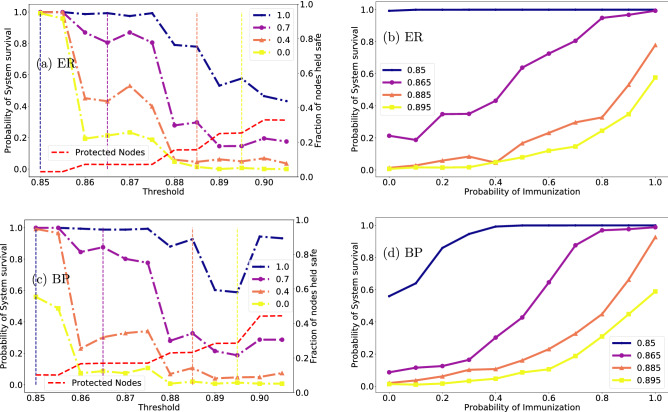
Comparison of the effect of probabilistic protection between several network structures. (**a**) ER network with 10,000 nodes and $\langle \hbox {k}\rangle =8$. The blue, purple, orange and yellow dotted-dashed lines represent a probability of 1, 0.7, 0.4 and 0 resp. for a chosen node to be protected. Blue and yellow lines represent full and no protection, respectively. The dashed red line shows the fraction of fully protected nodes with respect to the whole network. (**b**) Assuming four specific thresholds (vertical lines of matching colors to (**a**)) shown are the probability of system survival for all protection probabilities from 0 to 1; (**c**,**d**) same as (**a**,**b**) for the bipartite configuration described in “Network structure”.

## Models of cascading failures

Various approaches have been developed to simulate propagating failures in a network. One such approach sets a threshold for a node failure if its number of functional (non-failed) neighbors is below this threshold^27,28^. Since this resembles to the process of decomposing a network into its k-cores, this is also known as a k-core percolation. This model may be important in epidemiological applications, where the actual number of infected people with whom a person interacts is important. Another approach, called fractional threshold, is more relevant to settings such as opinion formation, infrastructures and financial and economic settings, measures the fraction of non-failed (or activated in the context of opinion spreading) neighbors of a node’s initial degree. The decision regarding whether or not the node will fail depends on the fractional threshold^11,29–31^. This process is illustrated in Fig. 1. The model stems, among others, from research in (1) opinion formation and (2) embracing of novelties. The assumption is that if *m* out of *k* friends adopt a certain behavior, the person connected with these friends will adopt it as well. The threshold for such process is not *m* but rather *m/k*. Hernce, a person with many friends will need, in absolute terms, more adopters in his or her vicinity in order to change the behavior. One of the fundamental studies of such fractional-threshold opinion propagation model is described by Watts in^29^. The condition that determines whether the network will collapse or survive given a minimal impact is central to this study. The cascade condition is derived using percolation arguments and is a first-order approximation for infinitesimal initial impact,

1 $$\begin{aligned} \sum _{k} k(k-1)\rho _kp_k>z, \end{aligned}$$

where k is the degree, $\rho _k$ is the degree-dependent probability distribution of the threshold, $p_k$ is the degree distribution of the network and z is the average degree.

Equation (1) is a good approximation that holds for an infinitesimal impact. A correction for finite impact has been developed in^32^, where a calculation to determine the eventual fraction of surviving nodes based on the connectivity and threshold distribution has been carried out. Gleeson and Cahalane^32^ show that the first order term in the expansion in a power series yields Watts’^29^ condition. Expanding to second order, a higher-importance term appears, governing the finite size cascading process. A detailed analysis of the onset and propagation of fractional cascading failure is developed by Di Muro et al.^33^ and is analyzed numerically in Supplementary Information  S1.

## Results

### Mitigation of cascading failure

Our main premise is that some nodes are more instrumental than others in propagating and exacerbating the failure process. Moreover, these nodes maybe identified based solely on their local environment. The algorithm is detailed in “Mitigation strategy” but its main idea is that we can determine such nodes and designate them for protection with ease. We find that our protection, even with partial information, is highly efficient in mitigating the cascading failure process. In order to be selected, the nodes must be fragile under a relevant failure mechanism, as per the definition in “Mitigation strategy”, and they need to have a sufficient amount of similarly fragile neighbors. Further specifications on connectivity of such nodes, once identified, help refine and reduce their amount. In essence, they correspond to the red nodes shown in Fig. 1. As a standard benchmark, we demonstrate our main results on an Erdos–Renyi (ER) network. Additionally, to be consistent with our later example of a real network, and inspired by^11^, a bipartite structure with one part’s degree distribution following a power law, and the second part’s—a random, Poisson distribution, is also analyzed. In the Supplementary we show additionally a scale-free (SF) network, as well as the more elaborate, but perhaps more common in life, interdependent pair of networks. The left panels of Fig. 2 demonstrate the behavior of the network survival vs. the system’s fragility, as expressed by the fractional threshold in the x-axis. The values on the x-axis specify the fraction of a node’s neighbors required to be active to survive (the failure mechanism is detailed in “Failure mechanisms”). The y-axis shows the probability of a system to survive. The different colors correspond to different protection probabilities (protection probability is detailed in “Randomness and lack of information”). The top (1.0) and bottom (0.0) curves in the left panel figures, corresponding to fully protected (1.0) and unprotected (0.0) networks, respectively. By fully protected we mean a node meeting the requirements specified in “Mitigation strategy” is indeed guaranteed survival. Reducing the probability of survival for protected nodes, will allow us to analyze the properties of the system under uncertainty (right panels of Fig. 2, “Randomness and lack of information”).

**Figure 3:**
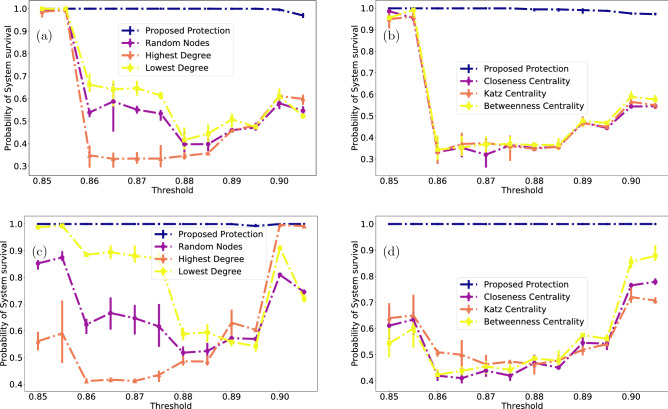
Different immunization approaches. (**a**) ER network with 10,000 nodes and $\langle \hbox {k}\rangle =8$. The blue, purple, orange and yellow dotted-dashed lines represent respectively, our strategy, a random selection of nodes, highest degree and lowest degree, (**c**) A bipartite network as described above under the same conditions as in (**a**); (**b**,**d**) follow the network structure of (**a**,**c**) resp. while comparing to centrality-based node selection, with purple, orange and yellow dashed-dotted lines corresponding to Closeness, Katz and Betweenness centrality, respectively.

Figure 2a shows the properties and behavior of the ER network. The yellow line (0.0) shows the evolution of a system with no external intervention. Below the critical threshold, just below 0.86 for the ER case, the system almost always survives. That is, repeating the experiment multiple times for randomly generated networks with the same macroscopic conditions almost always leads to the system remaining largely intact following the removal of a single node. As we increase the threshold, there is an abrupt transition to the system failing with high probability. The blue line, corresponding to keeping all nodes selected by our algorithm safe, shows the behavior of the system with external intervention. We now see that keeping all of our selected nodes safe from failure leads to significantly more resilient systems. It now remains almost always intact above the non-protected critical threshold, and even when it begins to fail—it does so much more rarely than in the unprotected case. In fact, as we show in Fig. 3, one can achieve a very high degree of stability by increasing the sensitivity of our selection algorithm (more on that in “Additional model parameters”). The intermediate lines, purple and orange, 0.7 and 0.4 resp., are obtained by holding our selected nodes safe with a given probability (70% and 40% here). This shows that even partially randomized protection allows us to noticeably increase the survival probability of our system. Finally, the dashed red line at the bottom of Fig. 2 shows the size of our selected set as a fraction of the system size (right axis). Reducing the protection probability would lead to a reduction of the protected set. We note here that both the basic cascading process (yellow lines) and effect of the mitigation strategy (blue lines) differ between the top and bottom of the left panel. While the bipartite network falls apart at lower thresholds (is more fragile), it is also more easily protected. The SF network in Supplementary Fig. S1a, for example, is much more highly protected using the same conditions as the ER one, leading to very resilient networks over a wide range of thresholds, even for 30% chance of failure for the selected set.

Importantly, near the critical threshold the protected nodes form a relatively small group, of the order of several percent of the entire network. A striking feature of the cascading process coupled with our immunization strategy is the lack of intermittent states of network health. That is, as shown in Supplementary Fig. S1, when a network collapses—it collapses completely (with the exception of the protected nodes which, by definition, survive). However, when the network survives—it survives almost entirely, with the possible exception of a small (compared to system size) set of nodes. We demonstrate that we can always select a set of nodes that is much smaller than the system size on one hand and ensures the system’s complete survival on the other. In this lies the novelty of our proposition. Such a result may be useful for systems where even partial failure is very costly, such as infrastructure networks. Complimentary, we provide an approach that allows decision makers to protect a network with a given probability, where potential loss is acceptable.

### Alternative mitigation benchmarks

Figure 3 and “Earlier simple strategies”, Centrality-based strategies show the advantages of the proposed mitigation approach compared to other existing strategies. Briefly, instead of finding nodes of importance and relying on them to save the network, we select the nodes that are most instrumental for avoiding spreading of the failure process and protect the system.

#### Earlier simple strategies

Following our analysis of different topologies and protection probabilities we wish to compare the effectiveness of our approach to other strategies. We begin with several trivial node selection algorithms. For each strategy we select the same amount of nodes as in our proposed strategy. The most basic “control group” is a random set. For that we choose from the nodes of the network a subset to be protected. Additionally, we choose the highest-degree nodes as the most reasonable candidates for protection. A plausible intuition behind this method is that selecting the most highly-connected nodes may help keep alive more of their neighbors. Then, following the understanding that the highest-degree nodes are not necessarily the ones propagating the cascade, we go to the other extreme and choose instead a set of nodes with the lowest degrees. Here the intuition may be that protecting the most vulnerable members of the network may ensure better survival. Figure 3a,c, and Supplemenatry Fig. S1a,c report the results on the topologies defined above. As mentioned in “Mitigation of cascading failure”, we increase the protected set here. That is done for two reasons: one is to demonstrate the possibility of definitive immunization for ER networks. The other is to demonstarte the advantages of out method to the ones shown in the figure. These are emphasized as we allow to increase the protected set in order to ensure survival under our strategy while still falling short for other mitigation method. Several interesting features surface in the analysis. For ER networks Fig. 3a, surprisingly, low degrees indeed provide better immunization than high ones (that under-perform even the random selection). The bipartite network Fig. 3c shows significant deviation from both ER and SF constituent types with an interesting crossover from a system apparently dominated by ER fragility for low thresholds to one dominated by SF fragility at the higher thresholds.

#### Centrality-based strategies

Here we evaluate other previous strategies of network immunization. We continue the line of reasoning that selecting nodes that are in some sense more important than others, may lead to a better outcome. To that end we employ several proposed methods of node selection. They are the Closeness centrality, Katz centrality and Betweenness centrality of nodes. Briefly, Closeness centrality is defined as the reciprocal of the distance between node *n* and all other reachable nodes; Katz centrality is a representative member of the eigenvalue centrality family of metrics where importance of a node is determined by the importance of its neighbors (i.e. the more significant the neighbors—the more significant the node); Betweenness of a node *n* is the ratio between the number of shortest paths between any two nodes that pass through *n* to all shortest paths between them. As with the simpler strategies, our approach is to rank all nodes in accordance with the selected centrality metric and select the same amount as proposed by our algorithm. The results are presented in the right panels of Fig. 3 for (b) ER network, (d) the bipartite construct, and Fig. S1b,d for a scale-free network and the interdependent cases respectively. As before, it can be seen that our proposed method outperforms the standard centrality metrics, highlighting the fact the nodes selected are not in particularly important on their own, as centrality would imply, but more important is their ability to facilitate failure spreading on the network. From this perspective, a reciprocal question may be posed, whether or not removing the selected nodes lead to an efficient network fragmentation, as discussed in, e.g.^34^, but as it is a broad topic on its own, we leave this question for further research.

## Methods

### Mitigation strategy

A reasonable approach to network immunization may begin by asking which node, if removed, causes the greatest damage to the system^23^. While this is insightful, it may not be sufficient. Indeed, Supplementary Sect. S1 and, specifically, Supplementary Fig. S1 shows the results of removing a single random node from a network. At the critical threshold even a very small random impact (e.g. removal of a single random node) is likely to cause a cascading failure. That means defining a node’s importance in terms of the damage it causes does not help from the mitigation perspective. We propose a complimentary approach, where we do not focus on how any single node removal affects the network, but rather, we ask which nodes are instrumental in *propagating* the cascade. Instrumental to our discussion is the notion of node fragility. There are many ways to understand this notion, so to avoid ambiguity we define the following:

#### Definition 1

Fragility—we define a fragile node under a failure mechanism, M, when the removal of a minimal relevant quantity, K of its links causes the node to fail.

As an example, for the fractional failure mechanism with threshold $\theta$, a node of degree *k* is fragile when a removal of a single edge will cause failure, i.e. $(k-1)/k < \theta$

Based on a very simple percolation argument we can conclude that in order for an impacted node to facilitate a cascade, it must meet two conditions:

1. It must be fragile as per the definition above.
2. It must have at least some neighbors which will also fail when impacted.

These conditions are intuitive, i.e. if a node does not fail upon a removal of a single neighbor, it is not very fragile and it will not be affected in the initial iteration of the cascade. If a node has less than two neighbors failing, it will not exacerbate the failure process (that is, at least locally, its failure will not increase the rate of failing nodes, since its branching factor is too low). These conditions become the two primary steps in our mitigation algorithm, as illustrated in Fig. 1: 
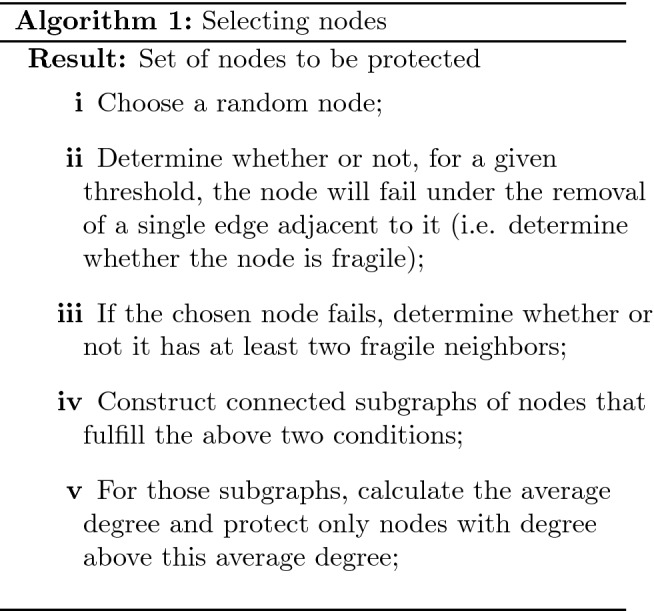


The last step is a heuristic which could be adjusted, as we describe below in “Additional model parameters”. At a first glance, there is nothing special about the selected nodes. However, note that high-degree nodes would not be selected for low thresholds as the high-degree nodes are not fragile until a very high threshold is reached. Low-degree nodes do not propagate enough damage and thus would not be selected for protection either. The seemingly common nodes, just weak enough to be fragile, yet connected enough to significantly propagate failure, would be the ones more likely to be protected. Importantly, although many nodes may appear to belong to this intermediate group of fragile and connected nodes, those complying with all our requirements are relatively few, thus creating a very cost-effective mitigation and protection strategy.

Having defined our strategy, we examine the consequence of securing the protected nodes. As discussed in “Results”, the different network structures lead to very different behaviors in terms of performance of the proposed mitigation strategy.

### Simulation setup

All our simulations are conducted on undirected, unweighted networks for tractability. Note, however, that the real network we test in “Application to European Banks’ sovereign debt exposures” is a weighted one. Our method is applicable without any modification. Network sizes were chosen typically of the order of 10 k nodes such that repeated realizations are feasible but finite size effects are negligible. For statistical validity, random setups are repeated at least 150 times (and up to 1,000 in some cases). For each iteration a new random graph is constructed via the appropriate methodology. A fractional threshold is then set for the nodes of the network. Nodes of degree 0 are removed to avoid degenerate cases. We initiate a cascade by removing a single node, holding the selected (protected) nodes “safe from failure”. The other nodes evolve as usual: at every iteration, nodes that are not within the protected set of nodes and have lost more neighbors than the fractional threshold permits, fail, potentially endangering all their neighbors. We proceed to iteratively remove all failed nodes until no updates are required to the remaining nodes and a steady state is reached.

### Network structure

Our main results are presented on an ER and bipartite networks as described above. Unlike many studied bipartite networks (citations, movies etc.), a network constructed of financial institutions on one hand and assets on another, is a realistic structure capable of transmitting a propagation of shock. Hence, removal of nodes and edges in such networks can cause significant, noticeable damage, mainly due to the network interconnectedness. As has been noted since Zipf^35–37^, firm sizes and incomes tend to follow a power-law distribution. The financial institutions have degrees distributed according to such a power law. The assets on the balance sheets, however, can be assumed to be randomly selected and thus distributed according to Poisson. In the Supplementary we show some corroborating evidence for this non-trivial structure. While we do not claim that it is an accurate representation of reality, it is both reasonable and instructive, as the behavior of such a network is qualitatively different from the standard networks.

To demonstrate the wide applicability, we report in the Supplementary the behavior of our algorithm when applied on a SF networks, as well as on an interdependent pair of networks^4,38^. These structures are standard in the field, corresponding to different real-world networks, and displaying different response to random and targeted failures. While SF networks are known to be more resilient to random failures due to the well-connected hubs, under the fractional threshold failure mechanism scale-free networks are more vulnerable compared to their ER counterparts with the same average degree^39^. The reason for this behavior lies in the network topology where the degree distribution is broad due to existence of hubs, while the majority of the nodes are poorly connected. Interdependent networks represent classes of infrastructure systems where critical resources are supplied by one network to the other and vice versa.

### Failure mechanisms

Several failure mechanisms are considered, the main one being fractional failure, where a node fails given an insufficient number of its neighbors survive. This model is relevant to many real-world systems such as finance, where a fraction of surviving neighbors may represent fraction of assets remaining after some default, or opinion formation where the fraction is that of friends adopting some position. A similar mechanism tested (but not reported, as it yields little additional insight) is the case of k-core failure, where instead of a *fraction* of surviving neighbors—a defined *number* of neighbors is needed to survive. This case may be more relevant for epidemics where the actual number (rather than fraction) of encountered individuals plays an important role. The main qualitative difference between the fractional and k-core is that nodes with degree below the initial threshold will fail without initial impact. Because of that, any sufficiently high threshold corresponds to a finite initial impact, as opposed to the infinitesimal one in the fractional threshold case. Another important mechanism is that of failure in interdependent networks. It is detailed in Supplementary Sect. S1.

### Randomness and lack of information

One strength of our approach is that it requires only information about a node’s fragility with respect to its failure mechanism (that is, we only need information about neighbors to decide whether or not a node fails, this information will also be sufficient to decide whether or not the node needs to be protected). But it is easy to envision a situation where not all nodes that pass the protection criterion as defined in “Mitigation strategy” can be protected. Such a situation may either be a result of incomplete information (not all local information is available for all nodes), insufficient resources (we need to protect M nodes but can afford to protect only N < M nodes), or as a means of maintaining ambiguity (a regulator may wish to signal an institution its survival is not guaranteed even if all conditions for protection are met, in order to promote prudence). In that case, we may ask how effective our mitigation approach would be if we could select only a subset of the nodes designated for protection. The answer to that question is seen in the right panel of Fig. 2. The colors match the vertical lines in the left panel and show the full details of varying the probability of protection. Thus, the solid blue line shows the network’s survival rate depending on probability of protection around the fragility threshold of the ER network, while the purple, orange and yellow lines go to higher and higher thresholds (i.e. areas of increased fragility). Here the x-axis traces the probability of immunization for a chosen protected node. Thus, whenever a node that belongs to a protected set is encountered, we randomly protect it with probability *p*. We observe in the right panel of Fig. 2 how different topologies respond to this probabilistic protection. Some differences of mitigation efficiency may be noted for the different networks. For example, for ER networks (Fig. 2b) above the critical threshold increasing the protection probability results in a gradual increase in mitigation efficacy. The initially more fragile bipartite network catches up with the ER one quickly as probability of immunization increases.

### Additional model parameters

Comparing the fully protected (blue) lines of the left panel of Fig. 2 to those of Fig. 3 one can clearly see that the survival probability is larger. That is due to the flexibility of our approach, where a broader subset of nodes is selected for protection yielding improved performance. One such flexible condition is step v in “Mitigation strategy” where different minimal degree of the induced subgraph may be defined. We could choose to protect more nodes by lowering the degree threshold in step v. The result would be that more nodes will be protected rendering a more stable network. However, since we assume that the protection of a node is not costless, we prefer to minimize the protected set, while still ensuring the survival of the network. Another alteration may be the minimal number of fragile neighbors required, where instead of two we may demand at least three for a smaller set, or less for better mitigation. Mapping out the full spectrum of parameters is left for further investigation. When the relation between failure mechanism and fragility is more complicated, such as the case of interdependent networks, we may define fragility in different manners. Those are described in Supplementary Sect. S2.

## Application to European Banks’ sovereign debt exposures

### Introduction

**Figure 4:**
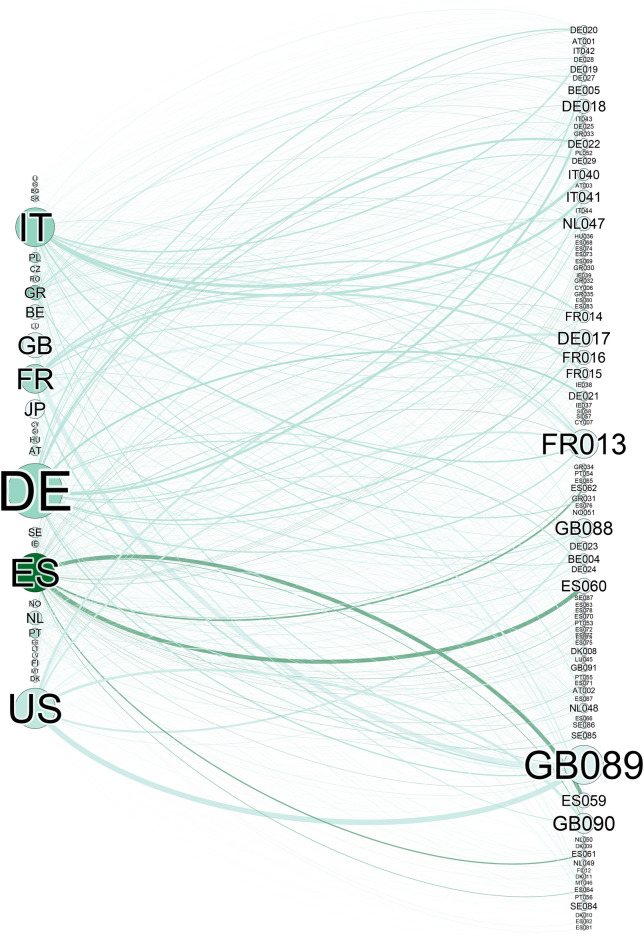
Financial network structure. The left side are various sovereign debt instruments. The right side are banks with their national belonging indicated by the leading letters. Node size indicates Dollar value; color—betweenness centrality. Edge width indicates investment size.

We now turn to an example of a real-world application of our suggested protection algorithm. As per the exposition, the data set we utilize for the test is the European banks and their holdings of Sovereign debt. The network is constructed by setting financial institutions and the debt instruments as nodes and joining by an edge a financial institution to a held debt. The network structure can be seen in Fig. 4.

### The data

Our data set consists of the sovereign debt exposure of close to 90 European banks recorded at 2011. The banks are anonymized, and the countries are abbreviated to two letter codes. The average bank holds $K_b = 12.7$ different assets while each asset is held by an average of $K_a = 35.8$ banks. This network is, of course, much smaller than the networks tested in the above discussed simulations. This limits our ability to discuss distributions, but will allow us, on the other hand, to measure the effect of different individual failures and how they are mitigated by our algorithm. Figure 4 visualizes the network structure, the left hand side being the sovereign debt instruments and the right—the banks holding them. Additional descriptive information can be seen in Supplementary Fig. S6—number of banks per country, the aggregated asset value of each country’s banks and holdings of individual banks with their degrees (number of assets held).

### Failure and mitigation in a real financial network

We now turn to the analysis of the failure process. While the model developed so far assumed integer degrees and unweighted edges, we wish to proceed here with more general assumptions. Now, instead of comparing the current degree to the initial one, we compare the value of the current holdings to the initial value.

The cascading failure process described here, follows Ref.^11^. We start with assuming a default of a sovereign debt, setting its value to zero. We then check whether the ratio of remaining banks’ holdings to initial holdings is above or below a specified threshold. If the ratio is below the threshold, the bank fails. Following that, the relative worth of the bank is removed from its holdings, thus the cascade continues.

Previously, we were agnostic regarding the origination of the shock. In this specific financial network analysis, we may expand our model by constructing immunization strategies depending on different types of potential failures. Alternatively, we can maintain the generality of the process by redefining the fragility of a node to be:

- Fragile under the failure of every neighbor: any failure in the portfolio may topple the bank
- Fragile under the failure of at least one neighbor: at least one debt failure in the portfolio may crash the bank (but not all—some failures may be absorbed by the bank)
- Fragile under some statistical measure—the mean, median or similar measure over the failure size crosses the threshold.

We apply the first assumption to the cascading failure process of the European Banks’ Sovereign Debt Holding network and find that the fraction of the network that needs to be protected is relatively small. Combined with the probabilistic approach from “Randomness and lack of information”, the set of protected nodes may decrease further.

**Figure 5:**
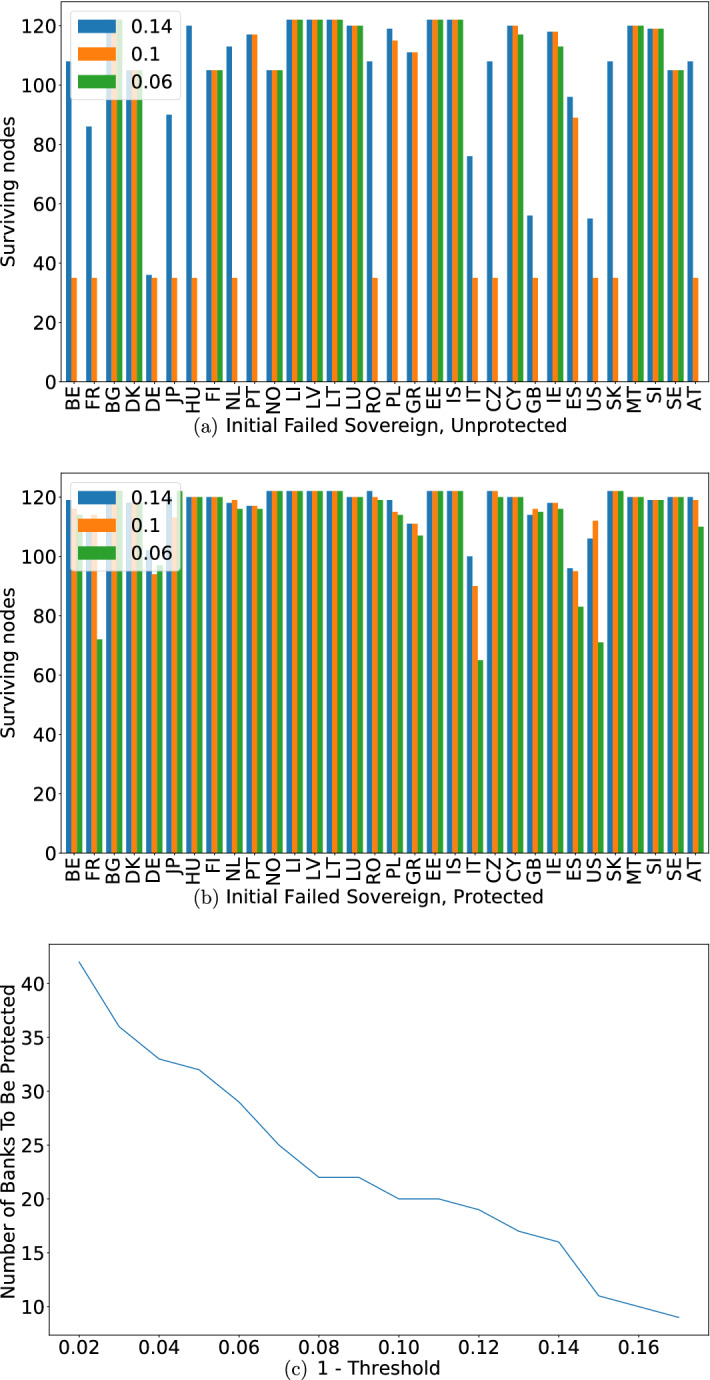
(**a**) Unprotected network. The horizontal axis shows the different failing debt instruments where, blue, orange and green bars correspond to (1-thresholds) of 14%, 10% and 6% resp. Height of the bar corresponds to the total number of surviving nodes (banks and assets). (**b**) The same network and failure process, but this time when applying our mitigation strategy showing a much higher survival rate for all initial impacts and thresholds tested. (**c**) Number of banks selected for protection per fragility threshold. As seen before, the more fragile the network—the more nodes are required for protection.

To assess the fragility of the system we start with a proposed initial failure and a survival threshold representing the required capital that banks need to hold on their balance sheets. To analyze the network we follow the following procedure: select a single sovereign debt and set its worth to zero (initial default). For a range of thresholds, perform the simulation to determine the progress (or lack-there-of) of a cascading failure. This allows us to map the fragility of the network given various combinations of initial conditions, in order to then assess the performance of our mitigation approach. Cascading failure results are presented in Fig. 5a. A few interesting points are worth mentioning here. One is that failure of some countries, such as Bulgaria (BG), Lichtenstein (LI) and Malta (MT) does not lead to a cascading failure even at high thresholds for the holdings as recorded (note that because in this section we are dealing with financial impact, we are more concerned with the fraction of value lost, shown as 1-*threshold*, as opposed to the fraction of surviving neighbors in previous sections). On the other hand, failures of countries such as Germany (DE), Italy (IT) and the United States (US), highly impact the network even at low thresholds. Lastly, there is a group of roughly 35 nodes (banks and assets) that form a group unaffected by the failure up to a certain threshold. Our next step, once we have examined the taxonomy of failure, is to apply our mitigation strategy. As mentioned above, the only adjustment to the original, unweighted strategy, is to set a node’s fragility in accordance with its sensitivity to the failure of any of its neighbors. Once fragility is defined, replacing step 2, we proceed with steps 3 through 5 as stated, and protect the selected nodes. In this case we choose to protect only banks, mirroring the Government bank bail-out process. It is also possible to protect debt, by guaranteeing a price floor to it. We turn to Fig 5b,c to analyze the results. To begin with, we note that for a relatively stable financial system, where an impact of 14% to the bank’s holdings is required to bring it down, protecting around fifteen financial institutions is enough to almost always spare a large fraction of the network, even in case of failure of financially vital sovereigns such as Germany, Japan (JP) or the United States. Even the cases where failure is severe, such as failures caused by the default of Italy or Spain (ES), we note that holding less than twenty institutions safe spares more than half the network (banks and debt instruments). Of course, as seen in the left panel of Fig. 2, increasing the fragility leads to a larger set of protected nodes. This case is no different, and having our network fail at a threshold of 6%, we now require protecting roughly a third of all financial institutions, but that, again, leads to an almost complete protection of the network aside for the aforementioned severe impacts. This is, of course, a very simplistic view of an intricate ecosystem. While our mitigation approach gives much promise, application to actual financial networks requires further analysis as discussed below.

## Discussion and conclusion

In this article we propose an algorithm that allows efficient mitigation of cascading failure processes on complex networks. The mitigation becomes possible due to the protection of selected nodes most potent at propagating and exacerbating failure. We have shown that proper selection is possible with minimal knowledge of the nodes’ local neighborhood and the failure mechanism. Our approach results in a very high probability of network survival without having a specific knowledge of the source of impact. We have tested various network structures and failure mechanisms and have obtained effective mitigation strategies. We also applied our approach to a real-world network of banks and assets and have shown it to perform well and succeed to significantly mitigate cascading failure following a hypothetical default on a government obligation. This work, however, leaves some open questions. As mentioned briefly above, some points warrant further research. For example, while our approach is successful in a wide variety of theoretical and some practical cases, and is intuitive—it lacks theoretical foundation. Development of a top-down theory may help refine some of the heuristics used in the definition of our strategy and elucidate their origins. Such a theory could help elucidate the effect of model parameters chosen “to work”. Additionally, though several network configurations and failure mechanisms were tested, both theoretical and real networks tend to have more intricacies. These include correlated networks, as well as those with rich community structures. Particularly in the case of financial networks, relations may be multiplex and time- and space-dependent. Also, in our study, we assume a simplifying uniformity of failure thresholds. We observe that initial impact has a strong effect on cascade propagation. While we do not assume tree-like networks, and in the case of our banks’ network we explicitly work with a network of very high degree, the feedback effects stemming from short cycles may have a non-trivial effect on the mitigation approach. Spontaneous healing of failed nodes or failure of new ones may take place (as a precursor to more general network dynamics). We also briefly mention the complementary question of network fragmentation based on the targeted attack of selected nodes. All these constitute questions to be explored and answered in order to refine our algorithm and expand its applicability. These questions notwithstanding, we believe the approach developed here may provide a useful tool for analysis and protection of real-world networks from events of catastrophic cascades.

## Supplementary information

Supplementary information
