# Supplementary material for: Mitigation of cascading failures in complex networks
Source: Sci Rep. 2020 Sep 30;10:16124. doi: 10.1038/s41598-020-72771-4 (PMC7528121; doi:10.1038/s41598-020-72771-4)
Supplement: Supplementary file 1 — Supplementary information [file 41598_2020_72771_MOESM1_ESM.pdf]

Mitigation of cascading failures in Complex Networks - Supplementary

Alex Smolyak[1], Orr Levy[1], Irena Vodenska[2], Sergey Buldyrev[3], Shlomo Havlin[1]

[1] Department of Physics, Bar-Ilan University, Ramat-Gan 52900, Israel;
[2] Department of Administrative Sciences, Metropolitan College, Boston University, 808 Commonwealth Avenue, Boston, MA 02215, USA;
[3] Department of Physics, Yeshiva University, 500 West 185th Street, New York, New York 10033, USA;

1. **Theoretical Background**

1.1. General topology solution and application to bipartite networks

Di Muro et al. [2] developed a framework for analytic calculations of the cascading failure processes taking place on interacting networks with varying inter- and intra-network degree distributions. The approach is general and allows calculating the evolution of cascading failures for a wide range of models, as well as for some analytical closed-form solutions for special cases. For the case of a bipartite network, we can modify the equations of Ref. [2] and from the interconnected network setting, keep only inter links, effectively reducing the setup to a bipartite network. We apply the analytical framework of the failure process to a bipartite network. Following Ref. [3], we define the generating functions as follows,

$$G_X(f) = \sum_{k=0}^{\infty} P_X(k) \sum_{j=0}^{k} r_X(k,j) f^j (1-f)^{k-j} C_k^j \tag{1}$$

$$H_X(f) = \sum_{k=0}^{\infty} k P_X(k)/\langle k \rangle \sum_{j=0}^{k} r_X(k,j+1) f^j (1-f)^{k-j-1} C_{k-1}^j, \tag{2}$$

where $P_X(k)$ is the degree distribution , r_X is the survival probability, and C_k^j is the binomial coefficient.
We then iterate Eqs. (1) and (2) until steady state is obtained:

$$\mu_{B1} = G_B(\mu_A),$$
$$\mu_B = H_B(\mu_A),$$
$$\mu_A = pH_A(\mu_B),$$
$$\mu_{A1} = pG_A(\mu_B).$$

Here, P_A and P_B are the degree distributions of networks A and B, r_A and r_B are the survival probabilities for A and B, μ_{A1} and μ_{B1} are the fraction of functional nodes at each step of the cascade, $1-\mu_A$, $1-\mu_B$ are the impacts required to produce the same effect at each step of the iteration, and $1-p$ is the fraction of removed nodes in the initial impact. Figures S1(a),(b) validate the analytic solution, Eqs. (1-1.1.), using simulations for a bipartite Erdos-Renyi (ER) random network, where each part's connectivity conforms to Poisson distribution with a fixed average degree and varying failure threshold with excellent agreement.
This approach is general and not limited to a specific degree distribution. We apply the above formalism on standard benchmark networks such as ER, scale free or others. Our survival function, r_X, can in principle be arbitrarily complicated and take into account various responses, as long as they can be locally defined, up to a restriction on monotonicity. For our simulations, following [4], we use the simple 0 or 1 function, if the current fraction of neighbors is below or above the set threshold respectively. Having defined those parameters we may readily obtain important information about the effect of cascading on our system. Figure S1 shows some analytical and simulation results of the cascading process. It highlights some valuable missing information. Specifically, while panels (a) and (b) support results obtained from the analytical analysis, panels (c) through (f) provide details unavailable from the iterative solution regarding the number of iterations (see further scaling relation in Fig. S2). The non-monotonous progress of the cascade allows us to determine (based on observable events) how far the cascading process has advanced at the time of observation (Fig. S1e,f). This knowledge allows us to propose appropriate actions to mitigate the deterioration of the system.

We study another important failure mechanism in the context of interdependent networks. As developed in [1, 5, 6] a pair of coupled networks, each with its topology of intra-layer links and with dependency links between the layers, will undergo cascading failures when a node fails given one of the two conditions: either it disconnects from the giant component or it loses its dependency link to the second network. The motivation for these two conditions is as follows: losing connection to the giant component may mean in many cases the de-facto loss of functionality. Taking the example in Buldyrev et al.[5], if a power plant or a communication router are disconnected from the network,

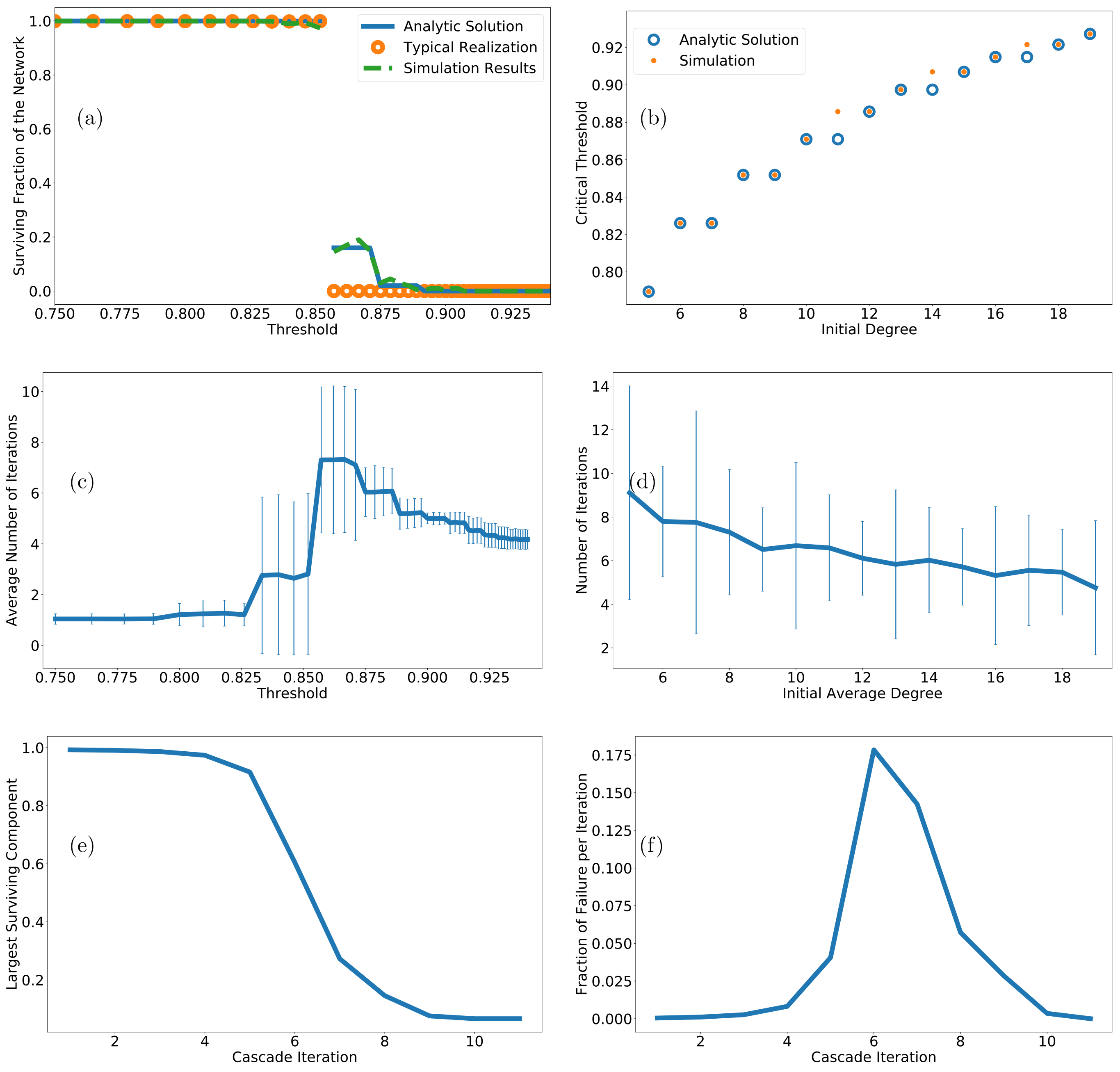

Figure S1: Demonstration of the fractional cascade process on a bipartite network of size of 8000 nodes, with ER degree distribution. a) Comparison between the analytic result (solid blue line) and numerical simulations for $\langle k \rangle = 8$, on a single realization (orange circles) and on average taken over 1000 realizations (dashed green line); b) Analytic and simulation results of the critical fractional threshold vs network's initial average degree; c) Average number of iterations per threshold for the setup in a). As shown in [1], failure takes the maximum number of iterations (around 8) to unfold at the critical threshold. Supplementary Fig. S2 expands on the scaling of the number of iterations; d) The number of iterations at the critical threshold for different initial average degree; e) The average remaining fraction of the network per iteration for $\langle k \rangle$=8 at the critical threshold; f) Average failure fraction per iteration.

even if they continue functioning themselves, they are no longer functional from the network perspective - i.e. power generated at the facility cannot reach clients and packets may not travel through the router. The second condition simply says that interdependence means a critical resource for functioning is supplied from a node in network A to a node in network B and vice-versa. Because of that, a failure of a node in one network would cause also the failure of its interdependent node in the other network. Conditions of one-to-one, full dependency have been alleviated in subsequent research [7], but we will proceed with the more fundamental fully dependent setting. While the first two types of failure: (i) K-core and (ii) Fractional failure models bear noticeable similarities, the failure mechanism of interdependent networks follows a very different path. We show that while we can have different definition of

node fragility, as described in Sec. 3.1., the overall mitigation approach proves that only knowledge of the (i) failure mechanism and the (ii) local structure are required in order to apply a successful immunization strategy.

2. **Behavior of Different Topologies**

Scale free networks bear more resemblance to real-world networks than ER ones, so testing our system on such cases of broad degree distribution is enlightening. Interdependent networks are yet another realistic and interesting case, that requires special attention due to the fact the cascading mechanism for interdependent networks differs markedly from the fractional or k-core threshold mechanism. As described in [5], nodes in interdependent networks fail because of one of two conditions: either they get disconnected from the giant component of their network (this may be the case of a major internet router being disconnected and thus ceasing to be functional as a part of the communications network), or due to the failure of a dependency link (such as the case of a power plant failing due to the absence of a critical resource like communication). Another adjustment to be made for interdependent networks is the initial impact needed to initiate a cascading failure. The average degree now does not affect not the critical threshold, but rather the initial impact, which is in principle a finite fraction of the network. In this scenario, keeping in mind that instead of threshold, the variable here is the fraction of nodes initially removed, due to finite size effects, we see noticeable probability of the networks collapsing even before the critical threshold of p_c=2.4554/⟨k⟩ [5]. Additionally, for interdependent networks, two different mitigation approaches are implemented, each corresponds to the global failure mechanism from a different perspective. In Fig. S3 a node is defined to be fragile if it is an articulation point, i.e. when its removal disconnects a non-zero set of nodes from the network [8], whereas in Fig. S4 global fragility is approximated by weak attachment to the network through low core-numbers. That is, a node is set to be fragile if its core number is low. Comparing the behavior of scale free and random networks we may conclude that while the scale free network is more fragile compared to the ER one, in the sense that it undergoes failure for a lower threshold, however, surprisingly, it is easier to protect and requires less than the full set of selected nodes. Moreover, above a rate of 0.7 protection probability the entire tested range of thresholds shows very high survival rates (Fig. S3b) compared to the ER network (Fig. 2b). The behavior of interdependent networks is somewhat different, among others due to the initial impact that is no longer infinitesimal. As can be seen in Figs. S3c and S3d our mitigation approach provides a much larger survival probability.

2.1. Alternative Protection

Different net types with alternative selection

For scale free networks S4a, even a random selection does a reasonable job for very low thresholds (as compared to the unprotected case in S3a) but the three simple approaches even out soon enough. Selecting enough high-degree nodes eventually pays off for very fragile systems. For the case of interdependent networks we limit the lowest-degree nodes to those with at least a single edge, otherwise it conflicts directly with the failure mechanism. Additionally, for the interdependent network in Fig. S4c we normalize the surviving nodes by the initial impact for ease of comparison to the other methods. As can be seen, here as well, protecting the weakest is most useful while the highest degree nodes provide the least protection. Centrality-based approaches (Fig. S4d) fare even worse than trivial node selection for the interdependent case

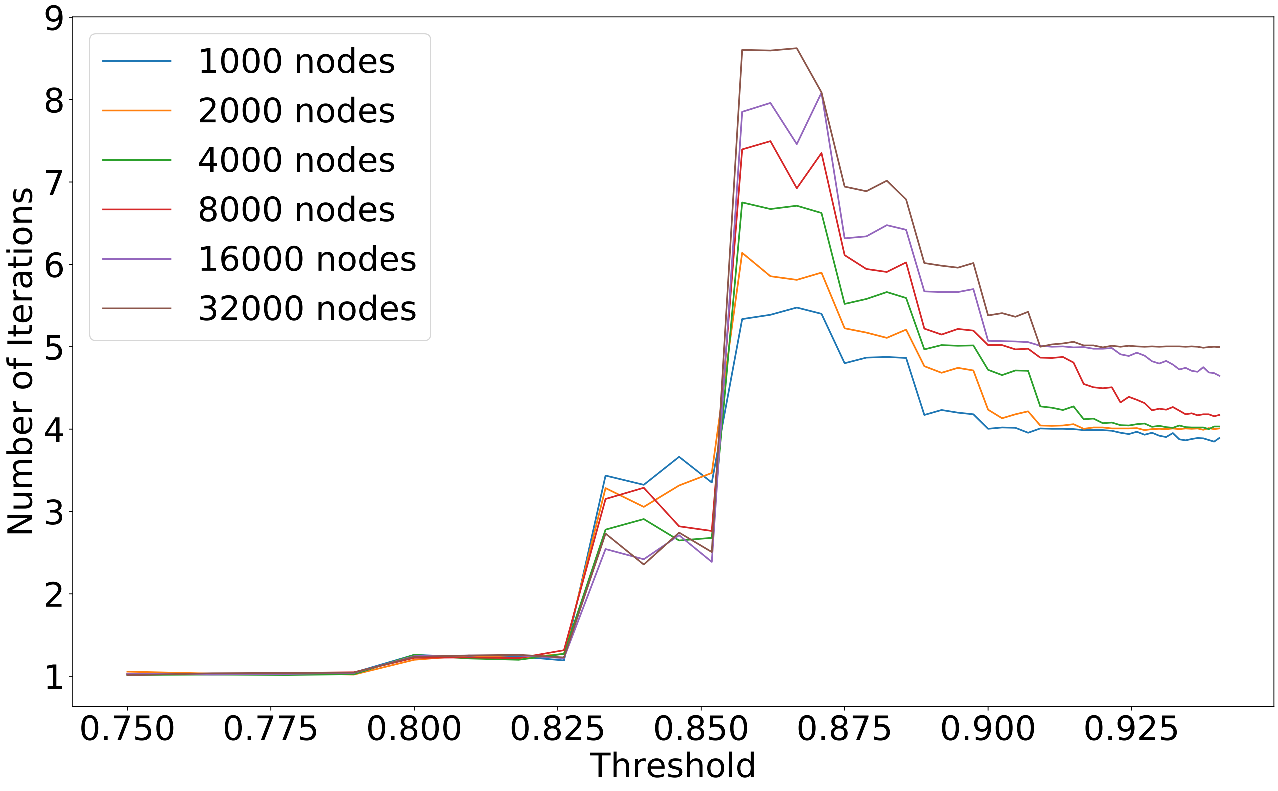

(a)

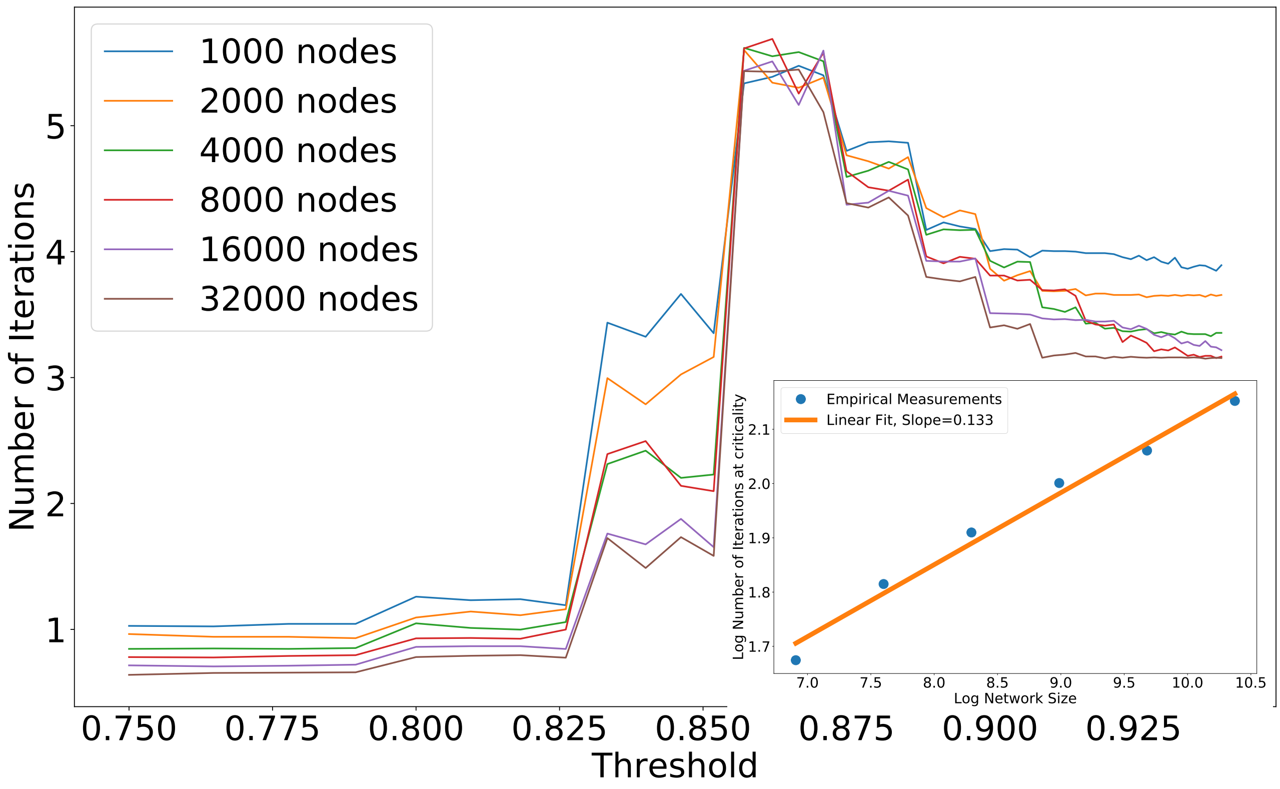

(b)

Figure S2: Scaling relations for number of iterations per threshold; Insert - Log-Log plot of network size vs Iterations with a linear fit, representing a power-law scaling

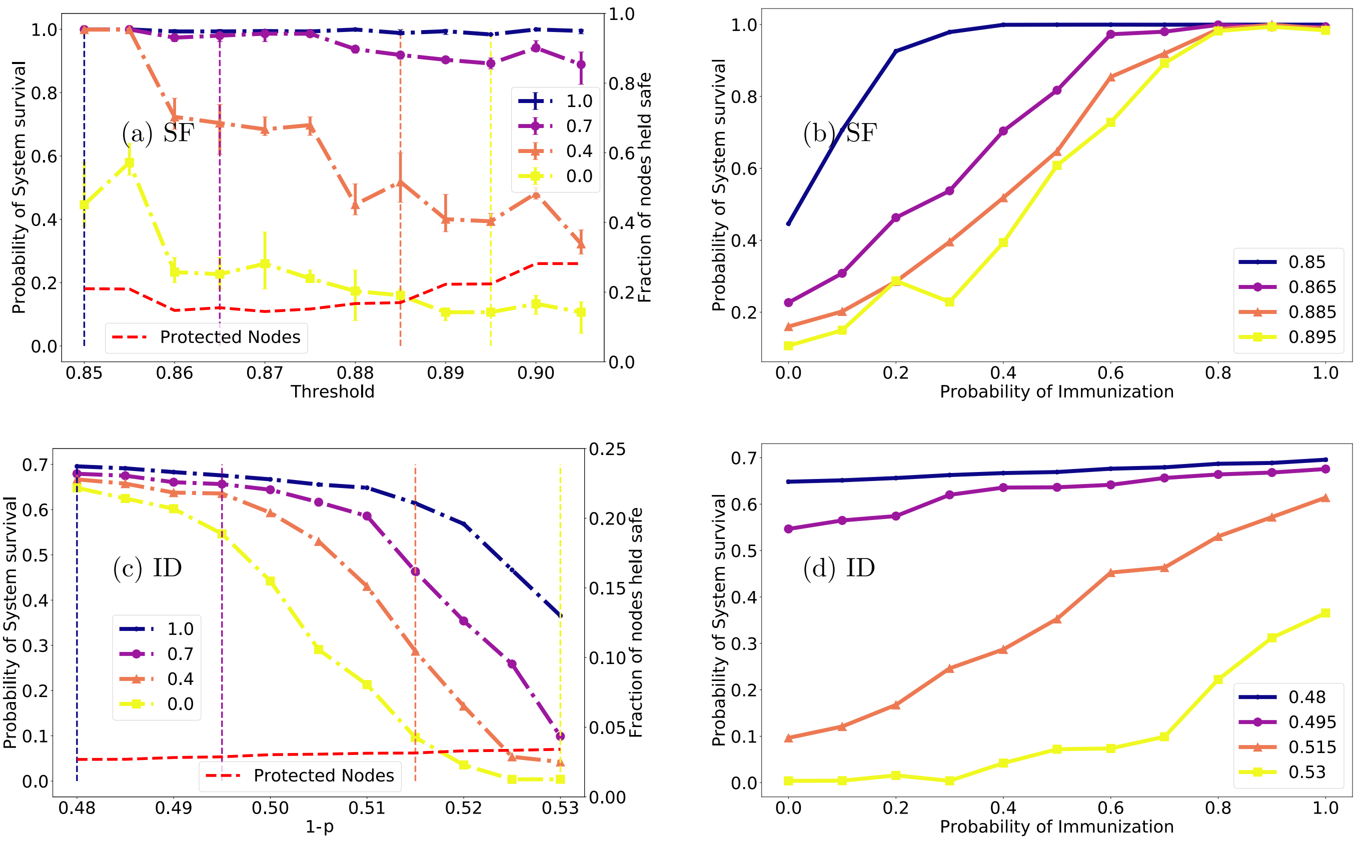

Figure S3: Comparison of the effect of probabilistic protection between additional network structures, complementing Fig. 2. a) a SF network with exponent 2.5 network with 10000 nodes and $\langle k \rangle$=8. Note that the critical threshold for a scale free network is lower, it fails where ER still holds. b) Probability of system survival for all protection probabilities from 0 to 1. Note the much better protection provided by the model where survival is ensured even at 30% chance of failure of protected nodes.; c-d) Same as a) and b) for interdependent networks with average degree $\langle k \rangle$=5, normalized by the initial impact.

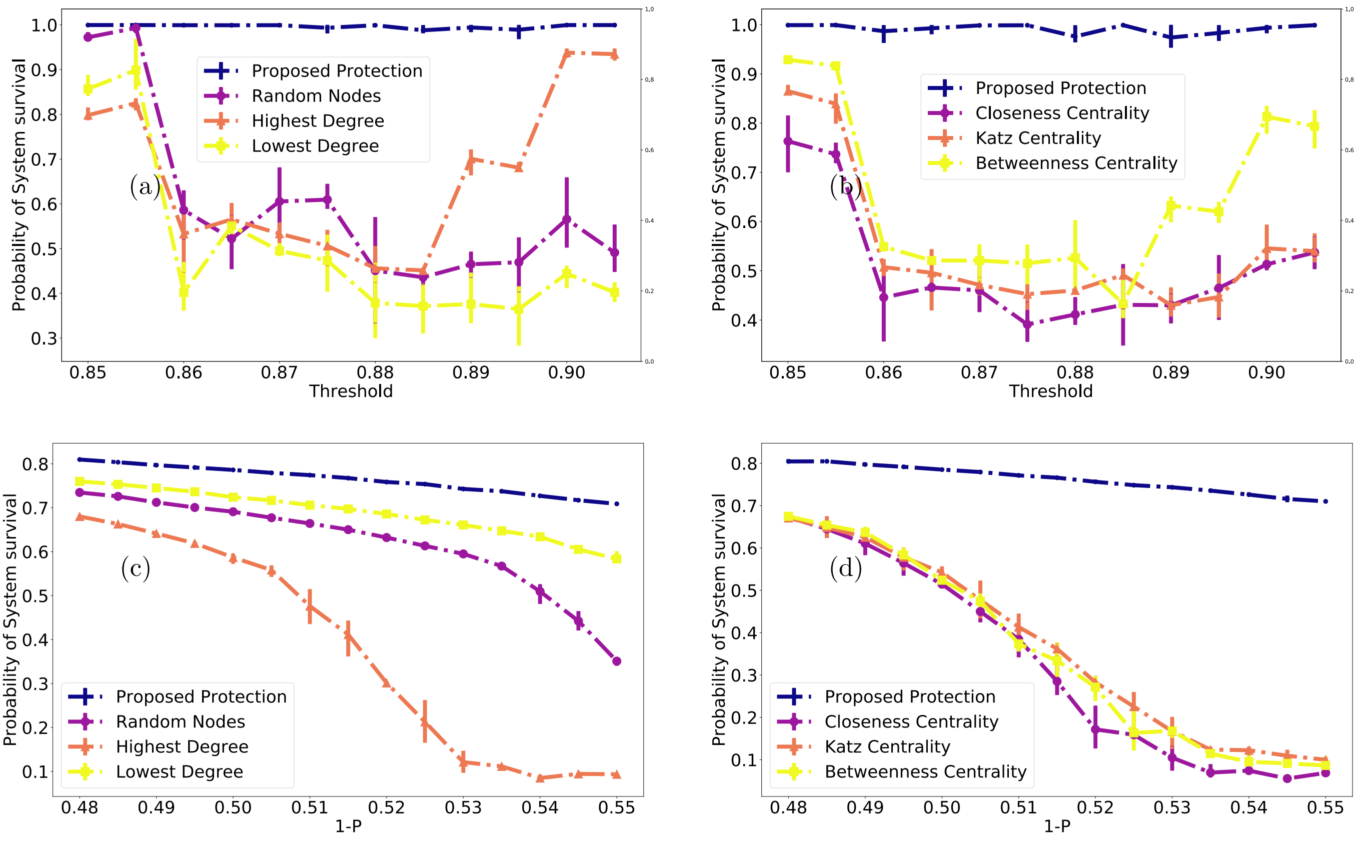

Figure S4: Different immunization approaches, complementing Fig. 3. a) SF network with 10000 nodes and ⟨k⟩=8. The blue, yellow, purple and orange dotted-dashed lines represent respectively, our strategy, a random selection of nodes, highest degree and lowest degree, c) Interdependent networks of average degree ⟨k⟩=5.; b) and d) follow the network structure of a) and c) resp. while comparing to centrality-based node selection, with yellow, purple and orange dashed-dotted lines corresponding to Closeness, Katz and Betweenness centrality respectively.

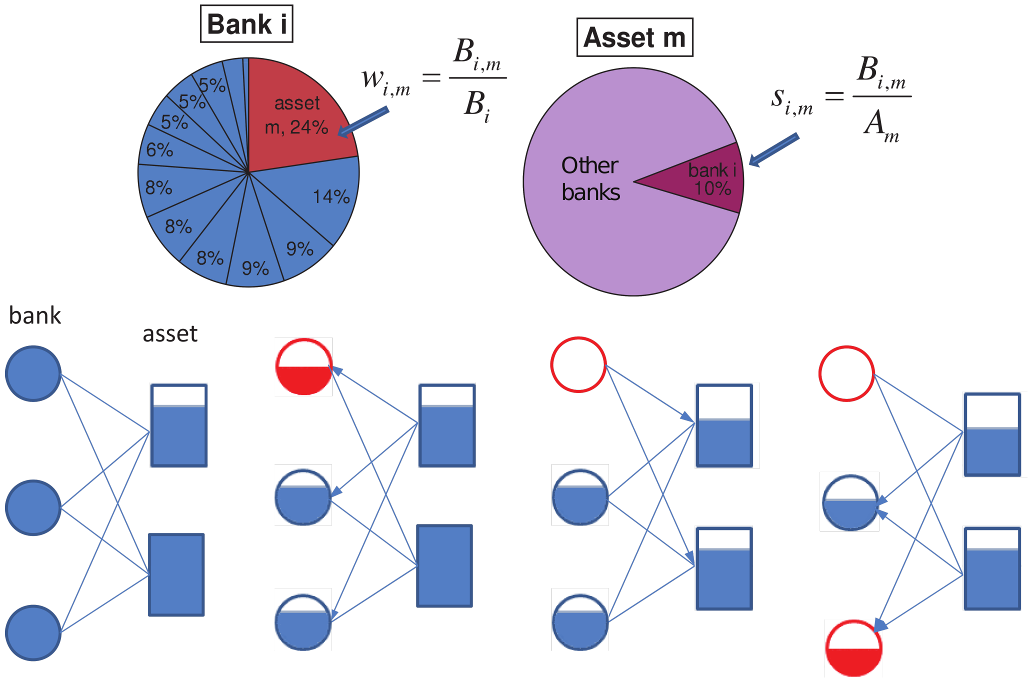

Figure S5: A fundamental model of failure propagation in a bipartite network. The fractional threshold is a natural extension for the failure of entities in financial systems. After [9]

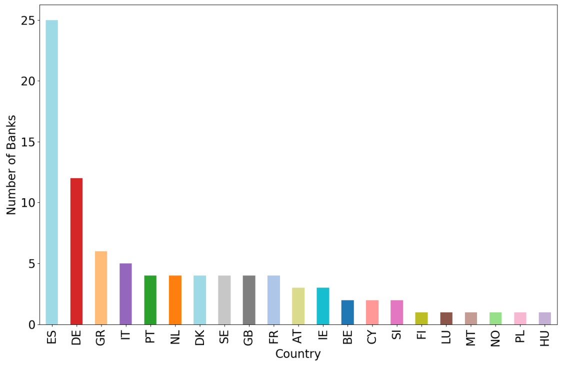

(a)

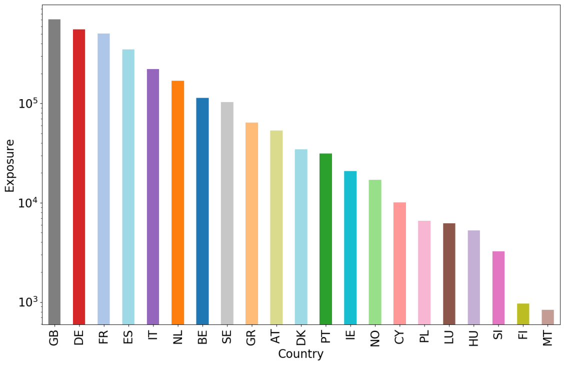

(b)

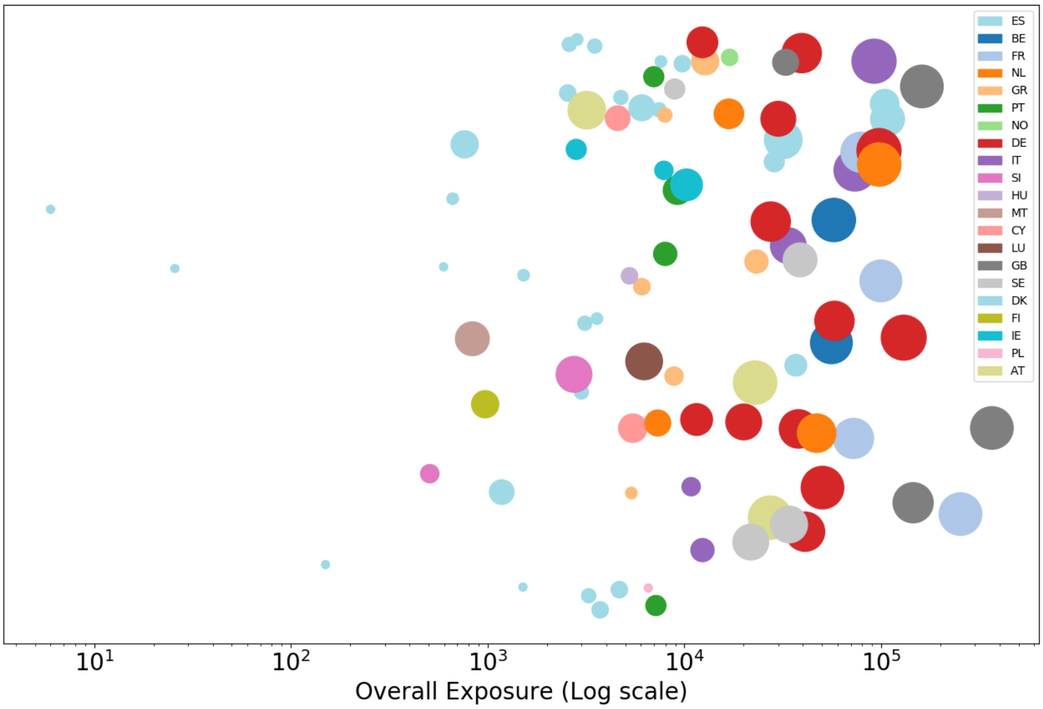

(c)

Figure S6: Seeing the debt: a) Number of banks per country; b) Aggregated exposure of a country's banks; c) Each Bank's exposure and degree. The degree (i.e. number of various sovereign debt held) is expressed via the size of the marker while the x-axis shows each bank's aggregated exposure. The vertical spacing is for convenience only and holds no meaning; Colors are consistent throughout the panel

References

1. Zhou, D. *et al.* Simultaneous first-and second-order percolation transitions in interdependent networks. *Physical Review E* **90,** 012803 (2014).
2. Di Muro, M. A., Valdez, L. D., Stanley, H. E., Buldyrev, S. V. & Braunstein, L. A. Insights into bootstrap percolation: Its equivalence with k-core percolation and the giant component. *Phys. Rev. E* **99,** 022311. `https://link.aps.org/doi/10.1103/PhysRevE.99.022311` (2 Feb. 2019).
3. Di Muro, M. A. *et al.* Cascading failures in interdependent networks with multiple supply-demand links and functionality thresholds. *Scientific reports* **7,** 1–10 (2017).
4. Watts, D. J. A simple model of global cascades on random networks. *Proceedings of the National Academy of Sciences* **99,** 5766–5771. ISSN: 0027-8424. eprint: `https://www.pnas.org/content/99/9/5766.full.pdf`. `https://www.pnas.org/content/99/9/5766` (2002).
5. Buldyrev, S. V., Parshani, R., Paul, G., Stanley, H. E. & Havlin, S. Catastrophic cascade of failures in interdependent networks. *Nature* **464,** 1025 (2010).
6. Gao, J., Buldyrev, S. V., Havlin, S. & Stanley, H. E. Robustness of a network of networks. *Physical Review Letters* **107,** 195701 (2011).
7. Parshani, R., Buldyrev, S. V. & Havlin, S. Interdependent Networks: Reducing the Coupling Strength Leads to a Change from a First to Second Order Percolation Transition. *Phys. Rev. Lett.* **105,** 048701. `https://link.aps.org/doi/10.1103/PhysRevLett.105.048701` (4 July 2010).
8. Tian, L., Bashan, A., Shi, D.-N. & Liu, Y.-Y. Articulation points in complex networks. *Nature communications* **8,** 14223 (2017).
9. Huang, X., Vodenska, I., Havlin, S. & Stanley, H. E. Cascading Failures in Bi-partite Graphs: Model for Systemic Risk Propagation. *Scientific Reports* **3,** 1219. arXiv: `1210.4973 [q-fin.GN]` (Feb. 2013).
